# Recent advances in defending the privacy attacks of large language models for healthcare applications: a concise review

**DOI:** 10.3389/frai.2026.1816692

**Published:** 2026-05-15

**Authors:** Rahma Aroua, Islem Kammoun, Mahmoud Aziz Louati, Donald A. Adjeroh, Tiehang Duan

**Affiliations:** 1 Department of Computer Science, College of Computing, Grand Valley State University, Allendale, MI, United States; 2 Lane Department of Computer Science and Electrical Engineering, West Virginia University, Morgantown, WV, United States

**Keywords:** differential privacy, healthcare informatics, HIPAA compliance, large language models, trustworthy AI

## Abstract

Large language models (LLMs) are increasingly adopted across healthcare applications, including clinical decision support and medical documentation systems. However, their deployment in medical settings raises significant privacy and security concerns due to the sensitivity of protected health information and stringent regulatory requirements. Recent studies have shown that LLM-based medical applications can cause medical data leakage through related API usages. This raises ethical concerns and threatens HIPAA (Health Insurance Portability and Accountability Act) compliance, blocking the trustworthy deployment of LLMs in the medical domain. This review examines emerging privacy attacks and the related defense approaches in LLMs with a special focus on healthcare applications. We organize the attack and defense approaches based on Secure AI Framework (SAIF), systematically covering vulnerabilities across the data, model, application and infrastructure layers. We performed detailed analysis on major classes of privacy attacks and further examined state-of-the-art defense mechanisms under realistic healthcare application scenarios. A key finding of this review is the persistent privacy-utility tradeoff: stronger privacy protection often leads to substantial degradation in clinical performance, which may render models unsuitable for mission-critical medical tasks. The healthcare related deployment of LLMs needs to be evaluated against clinical utility thresholds rather than generic language modeling metrics. We identify open challenges in evaluation, system-level deployment and regulatory verification, and outline research directions that balance clinical utility with regulatory compliance.

## 1 Introduction

The rapid advancement of Large Language Models (LLMs) has revolutionized artificial intelligence applications across diverse domains. In healthcare, models such as Med-PaLM (Singhal et al., 2023) and Clinical-BERT (Alsentzer et al., 2019) have demonstrated remarkable capabilities in clinical decision support, diagnosis assistance, and medical question answering (Chen, 2023). These models are promising to augment clinical workflows, and support more informed clinical decision making (Dennstädt et al., 2025).

However, the deployment of LLMs in medical settings raises critical privacy and security concerns that serve as significant barriers to adoption (Das et al., 2025; Yao et al., 2024). Recent work has demonstrated that LLMs can memorize and leak verbatim training data (Carlini et al., 2021), posing severe risks when models are trained on protected health information (PHI). The inadvertent exposure of patient data could violate HIPAA regulations, erode patient trust, and create legal liability for healthcare institutions (Dennstädt et al., 2025). Moreover, the regulatory landscape remains uncertain as existing frameworks struggle to address the unique challenges posed by generative AI in clinical contexts (Leiser et al., 2025).

Existing surveys on healthcare LLM privacy related topics adopt phase-based (pre-processing, fine-tuning, inference, and deployment) organization and analysis (Tahera et al., 2026; Leiser et al., 2025), here we systematically categorize attack surfaces using the Secure AI Framework (SAIF), providing component-layer analysis across the Data, Model, Application, and Infrastructure layers.

This review provides a comprehensive analysis of privacy and security challenges in LLMs, with particular emphasis on healthcare applications. We organize our discussion as follows: Section 2 examines privacy attacks targeting LLMs, Section 3 surveys the corresponding defense mechanisms, Section 4 discusses evaluation approaches, Section 5 addresses healthcare-specific challenges and regulatory requirements, and Section 6 identifies future research directions.

## 2 Privacy attacks on large language models

Privacy attacks exploit vulnerabilities across LLM system lifecycles to extract sensitive training data and model information. We organize attacks in alignment with the Secure AI Framework (SAIF) (Google, 2023), grouping risks by components include: Data, Model, Application, and Infrastructure. In this section, we categorize the attacks operating within these different components with detailed analysis on their functionality.

### 2.1 Model component

Training data extraction attacks aim to recover verbatim text from a language model's training data through direct querying of the model. Carlini et al. (2021) demonstrated that carefully crafted prompts can extract sensitive information from large language models. The proposed attack functions by generating text from the target model and comparing outputs to a reference model, and sequences with significantly lower perplexity in the target indicate memorization. They found that memorization can occur even without overfitting, and more importantly, rare and unique sequences are memorized at much higher rates than common text, making infrequent patterns particularly vulnerable to extraction.

Medical LLMs pose significant risks of training data extraction due to the sensitivity and uniqueness of clinical records. Studies show these models can memorize and reproduce protected health information, including patient-specific details (Li et al., 2026). Rare or unique sequences are particularly vulnerable (Carlini et al., 2021), which in clinical contexts often correspond to rare diseases or distinct patient trajectories, increasing re-identification risks (Jahan and Sun, 2025).

Membership inference attacks (MIA) aim to determine whether a specific data record was included in a model's training dataset. Fu et al. (2024) proposed to use self-prompted calibration, achieving AUC scores near 0.9 on membership inference attacks against fine-tuned large language models. The proposed method generates synthetic reference data and don't need to access original training data. However, Duan et al. (2024) conducted a comprehensive evaluation showing that membership inference attacks barely outperform random guessing on large-scale pre-trained language models, with near-random AUC scores for numerous tasks such as question answering, summarization, and classification.

This discrepancy reflects different evaluation contexts: Fu et al. (2024) tested small fine-tuned models while Duan et al. (2024) tested large pre-trained models, and the fact that vulnerability depends on model size, training regime, and specialization.

Membership inference vulnerability depends strongly on dataset size and model specialization. Models trained on smaller or homogeneous datasets are more prone to such attacks due to overfitting (Shokri et al., 2017), a common scenario in fine-tuned medical models (Li et al., 2026). In contrast, large pretrained models trained on diverse corpora tend to show lower attack success rates. This explains the gap between Fu et al. (2024) (AUC ≈ 0.9) and Duan et al. (2024) (AUC ≈ 0.5).

Model inversion attacks aim to reconstruct sensitive training examples by exploiting a model's output probabilities and confidence scores. Fredrikson et al. (2015) demonstrated this threat in the context of machine learning as a service, showing that by repeatedly querying a facial recognition system, attackers could reconstruct recognizable images belonging to the training set. Their work revealed that models leak information not just through their predictions, but also through the confidence within these predictions. Inversion attacks pose acute risks for medical LLMs as small fine-tuned models trained on limited clinical data may encode sufficient patient information to enable reconstruction. Systematic querying of medical chatbots could reveal patient notes, diagnoses, or treatment histories.

Attribute inference attacks (AIAs) allow adversaries to deduce sensitive traits like race or health status from model outputs or embeddings. This comes from overlearning, where models implicitly recognize sensitive features during primary learning tasks. Malicious servers can also use Active Attribute Inference (AAI) (Gomes et al., 2025) to purposefully modify neurons, encoding private patient data into *distinguishable footprints* on gradient updates. These attacks facilitate unauthorized profiling and discriminatory policies derived from clinical materials.

### 2.2 Data component

Gradient leakage in federated settings exploit a vulnerability in federated learning where participants share gradient updates instead of raw data. Despite appearing safe, these gradient updates can be inverted to reconstruct the original training data, undermining the privacy guarantees of distributed training. Hong et al. (2023) introduced the inversion influence function (I2F) framework to understand when and why gradient leakage succeeds. Their framework predicts reconstruction vulnerability based on data characteristics and model architecture. Deng et al. (2021)'s TAG method demonstrates that transformer gradient updates leak actual text tokens and sentences. Their work showed that even a single gradient update can reveal substantial portions of the training text, making gradient leakage a concrete threat for language models.

Data poisoning attack occurs when malicious actors intentionally seed misinformation into AI training datasets by hosting harmful content online for web crawlers to ingest. Alber et al. (2025) found that corrupting as little as 0.001% of training data can lead models to spread dangerous medical errors. Because compromised models still pass standard performance benchmarks, these attacks are extremely difficult to detect before the models are deployed.

### 2.3 Application component

Cross-context and cross-session leakage involve the unauthorized persistence or transfer of sensitive patient data between different users, sessions, or clinical tasks. This risk is particularly severe in shared medical workflows where multi-turn LLMs maintain contextual memory snapshots (KV caches) in GPU memory. If these snapshots are exposed during system debugging or hybrid-cloud scaling, they can reveal entire telemedicine transcripts or oncology notes to unauthorized personnel.

In multi-user clinical settings, weak session isolation can cause cross-context leakage between patients. For instance, a shared assistant may expose sensitive information from a prior interaction due to cached context. Foundation model analyses highlight risks of unintended data retention and prompt leakage (Bommasani et al., 2021). In healthcare, such disclosures can violate privacy regulations and undermine trust (Clusmann et al., 2023).

Retrieval privacy exfiltration healthcare Retrieval-Augmented Generation (RAG) systems extend the attack surface to the retrieval corpus, risking the exfiltration of sensitive intellectual property like clinical runbooks or patient registries Yao et al. (2026). Adaptive crawlers can systematically steal the corresponding knowledge base through multi-turn queries, a process facilitated by weak access controls and over-retrieval (high k depth). Additionally, embedding-based inversion attacks enable adversaries to reconstruct private narratives from hidden activations (Tahera et al., 2026).

### 2.4 Infrastructure component

Supply-chain attacks target pretrained models on repositories like Hugging Face. Attackers upload models that appear to perform normally on benchmarks but contain backdoors triggered by particular inputs, demographics, medical codes, or symptom phrases. Wang H. et al. (2025) embed such backdoors in pretrained embeddings, keeping poisoned representations statistically similar to clean ones, which allows backdoored models to pass validation. In healthcare, compromised models could misclassify cancer screenings for specific groups, suggest contraindicated medications for certain combinations of symptoms, or underestimate the severity of the disease in particular subpopulations.

Malicious LoRA adapters introduce deployment-stage supply-chain risk. Chen et al. (2026) propose CBA, a black-box, data-free backdoor attack on Low-Rank Adaptation (LoRA). adapters. Liu et al. (2025) introduce LoRATK, where a single backdoored adapter can infect many applications as users combine and share adapters. For medical LLMs, such adapters could inject false clinical information, while current repositories still lack strong provenance, signing, and automated backdoor checks (Google Cloud Security Team, 2024).

## 3 Defense mechanisms

**Figure 1:**
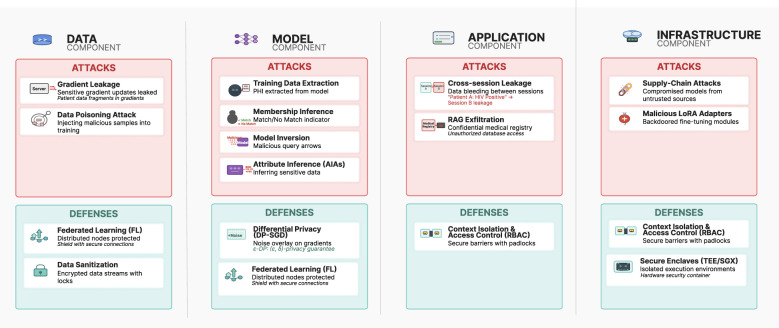
SAIF framework overview: mapping privacy attacks and defenses across the Data, Model, Application, and Infrastructure layers. Combined strategies (e.g., DP + FL) offer broader protection than single-mechanism defenses.

Major categories of defenses emerged in recent years including: differential privacy approaches that add calibrated noise to prevent information leakage, federated learning methods that enable collaborative training without centralizing data, together with access control related mechanisms. Figure 1 summarizes the mapping between privacy attacks and defense mechanisms across the SAIF framework layers, illustrating how each defense category addresses specific attack vectors. Defense of healthcare APIs must balance privacy with clinical utility, as excessive defense operations can degrade diagnostic accuracy to unsafe levels, a critical deployment challenge.

### 3.1 Differential privacy approaches

Differential privacy (DP) aims to prevent an attacker from determining whether any individual record was used during training (Du et al., 2025). Two primary approaches are DP-SGD (Wang L. et al., 2025), which adds calibrated noise to gradient computations, and the PATE framework (Papernot et al., 2017), which uses ensembles of teacher models trained on disjoint data and a noisy aggregation mechanism to train a student model that never directly accesses sensitive labels. Du et al. (2025) show that the effectiveness of DP-SGD depends on the choice of privacy budget and optimization configuration, and naive implementations that store per-sample gradients can incur prohibitive memory overhead (Wang L. et al., 2025). Boenisch et al. (2023) introduce individualized privacy budgets that allocate different privacy levels across training samples to mitigate this challenge.

### 3.2 Federated learning

Federated learning (FL) enables multiple parties to collaboratively train language models without sharing raw data. Each participant keeps their data local and only exchanges model updates, making FL particularly valuable in privacy-sensitive fields such as healthcare. Wang et al. (2024) introduced *FLoRA*, a framework that integrates Low-Rank Adaptation (LoRA) into federated settings to enable efficient fine-tuning by training lightweight adapter matrices rather than full model parameters. Complementary work has proposed benchmarks and aggregation schemes for federated LLM training (Bai et al., 2024; Ye et al., 2024).

Federated learning in healthcare is challenged by non-IID data across institutions. Variations in clinical settings, EHR systems, and data quality violate standard assumptions (Xu et al., 2021). Broader studies also highlight system heterogeneity and regulatory constraints (Kairouz et al., 2021), which complicate optimization and deployment of medical LLMs across diverse and regulated environments.

Despite these advances, FL systems still remain vulnerable to gradient leakage. Petrov (2024) showed that shared gradient updates can expose large parts of training data, with self-attention layers being especially susceptible to leakage. Recently, Shukla et al. (2025) demonstrated that combining FL with DP allows secure multi-institutional collaboration for breast cancer detection while ensuring compliance with HIPAA standards.

### 3.3 Runtime monitoring, guardrails, and input/output sanitization

Real-time auditing of prompts and outputs using embedding drift detection and PII classifiers prevent RAG exfiltration and cross-context leakage in deployed medical LLMs (Dong et al., 2025), monitoring anomalous query patterns before sensitive information is returned to users. Building on this detection capability, post-hoc guardrail frameworks operate as programmable, rule-based intermediaries that monitor and regulate real-time user interactions with deployed models. Within healthcare, this additional layer is essential for the sanitization of Patient Health Information (PHI), primarily achieved through deidentification, which removes direct identifiers such as medical record numbers and indirect identifiers such as zip codes to ensure compliance with HIPAA (Zhong et al., 2025). Advanced solutions such as Nvidia NeMo and Guardrails AI automate these protections by enforcing structural constraints, blocking unsafe content, and initiating corrective prompts (Dong et al., 2025).

### 3.4 Data sanitization and hardware-based protections

Securing medical LLMs requires protecting both training and inference. Data sanitization uses statistical outlier detection and influence functions to filter poisoned samples before training, mitigating data poisoning and backdoor risks in medical LLMs (Koh and Liang, 2017).

Hardware-based protections secure inference in untrusted environments. Trusted Execution Environments (TEEs) such as Intel Software Guard Extensions (SGX) (Widanage et al., 2021) provide hardware-isolated enclaves where memory remains encrypted outside the enclave and is only decrypted during processing, preserving confidentiality even if the operating system is compromised. For medical LLMs, this approach enables secure cloud-based inference where patient data can be processed without exposing PHI to cloud administrators, addressing key regulatory barriers to third-party LLM deployment in healthcare settings (Ng et al., 2025).

## 4 Evaluation metrics and benchmarks

Evaluating LLMs with standardized metrics help us translate abstract risks into measurable evidence about leakage, model utility and delployment readiness. In healthcare, this is especially important since even technically private models may be unacceptable if they degrade task performance below clinically safe thresholds. We examine these related metrics with analysis of healthcare-specific evaluation challenges.

### 4.1 Privacy metrics

Privacy metrics quantify the degree to which an LLM is protected against unauthorized disclosure of sensitive information under defined threat models (Yan et al., 2025). These metrics fall into two broad categories: formal metrics, which provide mechanism-level theoretical guarantees, and empirical metrics, which quantify observed leakage under specific attack models. In differential privacy, the formal guarantee is typically expressed through the privacy budget ε and, in approximate formulations, δ, where δ bounds the (usually very small) probability of a worst-case privacy failure, and smaller values indicate stronger protection but often at greater cost to model utility (Yan et al., 2025). By contrast, empirical evaluation depends on the attack class: membership inference attacks are commonly assessed with AUC or true-positive rate at fixed false-positive rates (Li et al., 2024a); training data extraction attacks with extraction success rate or canary exposure (Carlini et al., 2021); model inversion and gradient leakage attacks with reconstruction fidelity or re-identification success (Fredrikson et al., 2015; Hong et al., 2023). A robust evaluation should therefore report both formal privacy guarantees and attack-specific empirical leakage metrics.

### 4.2 Utility metrics

Utility metrics assess whether privacy-preserving LLMs remain effective for their intended healthcare applications despite the application of privacy-enhancing mechanisms. Common utility measures include perplexity, accuracy, and F1-score, which capture general language modeling and downstream task performance (Li et al., 2024a,c). In healthcare, however, utility evaluation must also reflect application-specific requirements in tasks such as clinical summarization, diagnostic decision support, patient-message drafting, retrieval-augmented question answering over medical records, and coding or documentation assistance (Amugongo et al., 2025). Accordingly, utility should be assessed using task-relevant measures such as diagnostic accuracy, factual consistency, clinical relevance, and decision-support reliability (Tahera et al., 2026), since even modest performance degradation may carry disproportionate practical consequences (Alber et al., 2025). The privacy–utility trade-off is reflected directly in these metrics, as stronger privacy protections often reduce task performance, making the key evaluation question not whether utility declines at all, but whether it falls below task-specific thresholds for acceptable clinical use. In healthcare applications, these thresholds are typically narrower than in general-domain settings because performance below clinically acceptable levels may render a system unsafe for deployment (Yan et al., 2025).

### 4.3 Evaluation frameworks and benchmarks

Recent frameworks have emerged to jointly evaluate privacy and utility. PrivLM-Bench (Li et al., 2024b) and LLM-PBE (Li et al., 2024b,c) provide structured approaches for assessing privacy protection across multiple attack types while measuring utility degradation. These frameworks enable researchers to compare privacy-preserving techniques under consistent evaluation protocols, facilitating progress in developing methods with favorable privacy-utility tradeoffs. Beyond these, Shen et al. (2026) introduced EAPrivacy, a benchmark for evaluating physical-world privacy awareness in LLM-powered agents such as embodied AI in hospitals, and reveals gaps in privacy reasoning for real-world scenarios.

### 4.4 Healthcare specific evaluation challenges

Healthcare deployment introduces unique evaluation requirements beyond standard privacy and utility metrics. Regulatory frameworks such as HIPAA and the General Data Protection Regulation (GDPR) mandate demonstrable safeguards for Protected Health Information (PHI), so privacy evaluations must be mapped to concrete compliance artifacts such as de-identification guarantees, residual re-identification risk, and auditability of data flows. In practice, this requires moving beyond abstract privacy scores toward task- and context-specific benchmarks that jointly measure (i) leakage under realistic attack models (e.g., extraction, membership inference, and RAG-based exfiltration on synthetic or sandboxed PHI), (ii) clinical performance on safety-critical tasks (diagnosis support, triage, prescribing), and (iii) regulatory alignment, including whether observed leakage would constitute a HIPAA or GDPR violation under existing enforcement guidance. Recent work on PHI-LLM evaluation and medical AI security frameworks highlights additional healthcare-specific gaps, including the lack of standardized PHI-aware benchmarks, limited transparency around “de-identification assessment” (Zhong et al., 2025), and the need for human-centered evaluation that captures clinician judgment and patient privacy expectations when models are used in real care settings (Ong et al., 2024).

## 5 Discussion: healthcare deployment, regulatory realities, and research gaps

### 5.1 Attack-surface differences for healthcare

Healthcare deployments of LLMs differ from general domains primarily in the *attack surface* created by (i) high-density protected health information (PHI), (ii) safety-critical workflow integration with minimum acceptable performance thresholds, and (iii) strict legal and institutional liability. Consequently, privacy and security risks should be analyzed as end-to-end system properties rather than model-only artifacts. In practice, all system layers including data, model, application, and infrastructure contribute materially to PHI exposure and must be evaluated jointly (Dennstädt et al., 2025; Patel, 2025).

### 5.2 Deployment implications: architectural risk hotspots

In deployed healthcare LLM systems, PHI leakage is driven less by model internals and more by system integration within clinical workflows. In practice, privacy risks concentrate in three architectural hotspots: cross-context handling under prompt manipulation, retrieval-augmented generation (RAG) pipelines, and reliance on opaque third-party services (Dennstädt et al., 2025). These hotspots define the primary failure modes of real-world deployments and motivate targeted architectural safeguards.

#### 5.2.1 Risk hotspot 1: prompt injection and cross-context leakage

Clinical deployments involve multiple users, sessions, shared tools, and internal data access, increasing exposure to prompt injection and context manipulation. In healthcare, context boundaries extend beyond sessions to include patient identity, clinical encounter, user role, and institutional scope. Ensuring isolation across these dimensions is therefore critical. Mitigations include strict context isolation, input sanitization, policy enforcement, and least-privilege tool access, particularly restricting queries to the active patient context (Dennstädt et al., 2025).

#### 5.2.2 Risk hotspot 2: RAG as mitigation and new surface

While RAG reduces reliance on memorized model parameters, it introduces new privacy risks, including mis-scoped retrieval, permission violations, and over-disclosure from heterogeneous or untrusted sources (Mathai, 2025). In practice, these failures often stem from misconfigured access controls rather than model behavior. In clinical settings, retrieval authorization becomes the primary privacy boundary, as post-generation filtering cannot prevent exposure once sensitive data enters the prompt. Mitigations include fine-grained access control, minimal-disclosure retrieval, audited knowledge bases, and provenance-aware outputs to support auditing (Mathai, 2025; Patel, 2025).

#### 5.2.3 Risk hotspot 3: vendor lock-in and limited auditability

Reliance on third-party or opaque systems limits visibility into data handling and privacy guarantees. This constrains verification and increases institutional risk. Mitigations include enforceable data governance agreements, comprehensive audit logging, retention controls, and architectural choices that preserve observability, such as client-side monitoring and policy enforcement where feasible (Dennstädt et al., 2025; Patel, 2025).

### 5.3 Regulatory realities reframed as operational requirements

Rather than surveying regulatory frameworks, we summarize operational requirements that support compliance-oriented risk management in healthcare deployments (Dennstädt et al., 2025; European Data Protection Board, 2025):

- Data flow transparency: document where PHI enters, is stored, processed, and exits the system (including tools and third-party services) (European Data Protection Board, 2025).
- Access control and least privilege: enforce role-based permissions for data sources, tools/agents, and outputs (European Data Protection Board, 2025; Patel, 2025).
- Retention limits (including logs/telemetry): minimize and time-bound storage of prompts, retrieved text, outputs, and associated metadata (Dennstädt et al., 2025).
- Audit logging: maintain query, retrieval, and access logs sufficient for investigation and compliance reporting (Dennstädt et al., 2025; Leiser et al., 2025).
- Isolation boundaries: tenant/session isolation to prevent cross-user leakage in shared deployments (Patel, 2025).
- Incident response readiness: monitoring, alerting, and response procedures tailored to LLM failure modes (e.g., unexpected disclosure patterns and canary exposure) (Leiser et al., 2025; Patel, 2025).
- Third-party governance: contractual controls (e.g., business associate agreements) and provider data-handling assurances (e.g., defined retention periods and restrictions on secondary use) (Dennstädt et al., 2025).

A recurring gap remains: while these policies can be specified, verification is technically challenging when model weights and provider-side processing are opaque (Dennstädt et al., 2025; Leiser et al., 2025).

### 5.4 Prioritized agenda: gaps → directions → evaluation

Table 1 summarizes a compact research-and-deployment roadmap that links identified gaps to concrete directions and the corresponding evaluation approaches. Specifically, rows 2 and 5 operationalize the RAG and cross-context leakage hotspots discussed above.

**Table 1:** Comprehensive roadmap for secure medical LLM deployment across SAIF framework layers.

SAIF layer	Current gap	Proposed solution	Evaluation method	Priority	Key references
Model layer	Memorization of rare PHI	Document-level DP; utility recovery via self-distillation	Extraction rate; canary tests	High	Ngong et al., 2025
	MIA on fine-tuned models	Adaptive DP budgets; regularization	AUC; false positives	Medium	Fu et al., 2024; Duan et al., 2024
Data layer	Gradient leakage in FL	FL + DP; secure aggregation	Reconstruction fidelity	High	Xu et al., 2023; Petrov, 2024
	De-identification failure	Context-aware anonymization	Re-identification rate	Medium	–
Application layer	RAG permission failures	Permissioned retrieval; minimal disclosure	Permission violations; PHI disclosure	Critical	Amugongo et al., 2025
	Cross-session leakage	Context isolation; session-bound memory	Leakage tests; injection results	Critical	Lee et al., 2025
	Prompt injection	Input sanitization; output validation	Injection success rate	High	Clusmann et al., 2025
Infrastructure layer	Third-party auditability	Audit logging; compliance validation	Logging completeness; coverage	High	Dennstädt et al., 2025
	Supply-chain backdoors	Model verification; cryptographic signing	Backdoor detection rate	Medium	Wang H. et al., 2025; Liu et al., 2025
Cross-cutting	Privacy-utility tradeoff	Adaptive budgets; clinical constraints	Clinical performance vs. ε	Critical	Yan et al., 2025
	Lack of benchmarks	PHI-specific metrics; medical frameworks	Benchmark adoption	High	Li et al., 2024b,c

It highlights that a meaningful roadmap for secure medical LLMs must connect gaps, candidate solutions, and concrete evaluation signals rather than treating them in isolation. Rows at the application layer make this especially clear: RAG permission failures and cross-session leakage directly instantiate the deployment “hotspots” discussed in Section 5.2, but the table refines them into testable outcomes such as permission-violation rates, PHI-disclosure rates, and cross-context leakage tests. At the same time, cross-cutting rows (privacy-utility trade-off and lack of benchmarks) emphasize that architectural fixes are insufficient unless they are tied to clinically grounded performance thresholds and PHI-aware metrics that can be adopted across institutions. Taken together, the roadmap therefore suggests a prioritized agenda: harden the most critical hotspots (RAG and cross-context leakage), embed them into repeatable red-teaming and regression tests, and iteratively expand evaluation coverage toward standardized, shareable benchmarks for privacy-preserving medical LLMs.

## 6 Conclusion

This review surveyed privacy and security risks in medical large language models (LLMs) through the lens of the Secure AI Framework (SAIF), mapping attacks and defenses across the Data, Model, Application, and Infrastructure layers. A central conclusion is that deployments of the defense approaches face a *structural* privacy-utility tension. We pinpointed three major architectural risk areas where protected health information (PHI) leaks are most common: prompt injection, RAG permission failures, and vendor opacity. Looking forward, significant progress is needed in the following directions. First, defense mechanisms need substantial technical development with concrete implementations on top of existing research. Second, we need to develop standardized evaluation metrics for healthcare-related applications. Finally, we need more research on how these attack and defense methods actually perform in real-world clinical settings. Addressing these needs helps us develop trustworthy medical LLMs that protect patient privacy while ensuring clinical usefulness.
